# Functional Outcomes and Patient Satisfaction in Kinematic vs Mechanical Alignment Total Knee Arthroplasty: A Systematic Review

**DOI:** 10.7759/cureus.93939

**Published:** 2025-10-06

**Authors:** Wael A Rahman, Khalid Y Muqri, Hussam M Suhail, Mohammed M Shajri, Yazan Z Omar, Mohammed A Alahmari, Abdulrhman A Hakami, Rawan S Alotaibi, Nasser H Alowaimer, Mohammed S Alrehaili

**Affiliations:** 1 Department of Orthopedic Surgery, Prince Mohammed Bin Abdulaziz Hospital, Riyadh, SAU; 2 Department of Orthopedic Surgery, King Fahad Medical City, Riyadh, SAU; 3 College of Medicine, Jazan University, Jazan, SAU; 4 Faculty of Medicine, King Abdulaziz University, Jeddah, SAU; 5 College of Medicine, Ibn Sina College of Medicine, Jeddah, SAU; 6 College of Medicine, Imam Mohammad Ibn Saud Islamic University, Riyadh, SAU; 7 Faculty of Medicine, Umm Al-Qura University, Makkah, SAU

**Keywords:** functional outcomes, implant survival, joint awareness, kinematic alignment, mechanical alignment, osteoarthritis, patient satisfaction, revision rates, systematic review, total knee arthroplasty

## Abstract

Total knee arthroplasty (TKA) is a standard treatment for end-stage osteoarthritis, yet up to 20% of patients remain dissatisfied. Alignment strategy is a critical determinant of outcomes. Mechanical alignment (MA) has long been the conventional approach, while kinematic alignment (KA) has gained attention for its potential to restore native knee anatomy and improve patient-centered results. This systematic review compared functional outcomes, patient satisfaction, and safety between KA and MA in primary TKA. A comprehensive search of PubMed, Web of Science (WOS), Scopus, and the Cochrane Central Register of Controlled Trials (CENTRAL) through August 2025 identified randomized controlled trials and comparative cohort studies reporting functional outcomes, patient-reported measures, satisfaction, or revision rates. Methodological quality was appraised using the Modified Downs and Black checklist. Fourteen studies met the inclusion criteria, representing diverse populations and designs with moderate-to-high methodological quality (scores 22-25/28). KA demonstrated small-to-moderate short- to mid-term improvements in Oxford Knee Score (OKS), Western Ontario and McMaster Universities Osteoarthritis Index (WOMAC), Knee Society Score (KSS), Forgotten Joint Score (FJS), and early flexion range of motion, particularly in varus phenotypes and when joint line orientation was preserved. It was also associated with fewer soft-tissue releases and improved intraoperative balance. However, long-term follow-up showed convergence of outcomes, with equivalent survivorship and complication rates between KA and MA. Patient satisfaction trends favored KA during early recovery, though pooled evidence did not demonstrate consistent superiority. KA appears to provide meaningful short- to mid-term advantages in functional recovery, joint awareness, and satisfaction without compromising implant survival or safety, especially in varus-aligned patients when applied within restricted boundaries. Nevertheless, heterogeneity in surgical techniques and outcome reporting highlights the need for large, phenotype-stratified randomized trials with long-term follow-up to establish optimal alignment strategies in TKA.

## Introduction and background

Knee osteoarthritis is a leading cause of pain, disability, and reduced quality of life worldwide. When conservative management fails, total knee arthroplasty (TKA) is one of the most effective and widely performed orthopedic procedures [1]. Demand for TKA is projected to rise substantially due to aging populations and increasing obesity rates. Despite improved implant survival, up to 20% of patients remain dissatisfied, emphasizing the need to refine surgical strategies to optimize function and satisfaction [2].

Mechanical alignment (MA) has long been the standard, positioning components perpendicular to the mechanical axis to restore a neutral hip-knee-ankle angle [3]. This is intended to ensure uniform load distribution and reduce implant wear [4]. However, MA often requires extensive soft-tissue releases and alters native anatomy, which may compromise kinematics and outcomes [2].

Kinematic alignment (KA) has been proposed as an alternative, aiming to restore constitutional limb alignment and joint line orientation [1]. By resurfacing in line with pre-arthritic anatomy, KA preserves ligament tension and minimizes soft-tissue releases [2]. Early reports suggest benefits in range of motion, functional recovery, and perceived “naturalness,” though concerns remain regarding implant longevity in outlier alignments [2].

Patient-reported outcome measures (PROMs) such as the Oxford Knee Score (OKS), Western Ontario and McMaster Universities Osteoarthritis Index (WOMAC), Knee Society Score (KSS), Knee Injury and Osteoarthritis Outcome Score (KOOS), and Forgotten Joint Score (FJS) are widely used to assess pain, function, and joint awareness. Some randomized and cohort studies show higher scores and faster recovery with KA [5,6], while others report no significant differences [2].

Patient satisfaction is increasingly recognized as a critical endpoint. Dissatisfaction may persist despite technically successful surgery due to residual pain, instability, or unnatural kinematics [7]. Some evidence suggests KA may improve satisfaction, particularly in varus phenotypes, by preserving joint line obliquity and ligament balance [8]. However, conflicting results and limited long-term data remain. Given these uncertainties, this systematic review synthesizes evidence on functional outcomes, satisfaction, and safety of KA versus MA in primary TKA.

## Review

Methods

Literature Search Strategy

This systematic review adhered to the Preferred Reporting Items for Systematic Reviews and Meta-Analyses (PRISMA) guidelines [9]. A comprehensive search was conducted across four major electronic databases, PubMed, Web of Science (WOS), Scopus, and the Cochrane Central Register of Controlled Trials (CENTRAL), from inception to August 20, 2025. A combination of controlled vocabulary and free-text terms was used, including (“total knee arthroplasty” OR “total knee replacement” OR “TKA”) AND (“kinematic alignment” OR “restricted kinematic alignment” OR “inverse kinematic alignment”) AND (“mechanical alignment” OR “conventional alignment”).

Eligibility Criteria

Selection criteria were defined using the population, intervention, comparison, outcome, and study design (PICOS) framework [10]. We included English-language randomized controlled trials and comparative cohort studies that enrolled adult patients undergoing primary TKA for osteoarthritis, evaluated kinematic alignment (KA), including restricted or inverse KA techniques, compared outcomes against mechanical alignment (MA), and reported at least one relevant outcome measure such as functional scores (OKS, KSS, WOMAC, KOOS, FJS, range of motion), patient satisfaction, radiographic outcomes, or complication/revision rates. Exclusion criteria were revision TKA, unicompartmental knee arthroplasty, case reports, case series with fewer than 10 patients, reviews, technical notes, conference abstracts, and non-English publications.

Study Selection

Two reviewers independently screened titles and abstracts of all retrieved records against the eligibility criteria. Studies not meeting the inclusion criteria were excluded at this stage. Full texts of potentially eligible articles were then assessed in detail. Any disagreements were resolved by discussion, and when necessary, a third reviewer was consulted to achieve consensus.

Data Extraction

Full texts of the included studies were analyzed in detail, and the following data were extracted using a standardized form: author and year of publication, country of study, study design, sample size, demographic characteristics (age, gender, BMI), surgical technique (kinematic versus mechanical alignment), type of prosthesis, outcome measures (OKS, KSS, WOMAC, KOOS, FJS, range of motion, satisfaction scores, radiographic findings), and key results including complications or revision rates. Data extraction was performed independently by two reviewers, with disagreements resolved through discussion or referral to a third reviewer.

Quality Appraisal

The methodological quality of included studies was independently assessed by two reviewers using the Downs and Black checklist for clinical trials [11]. This tool evaluates 27 items across four domains: reporting, external validity, internal validity (bias and confounding), and power. Total scores range from 0 to 28, with studies classified as excellent (26-28), good (20-25), fair (15-19), or poor (≤14). Discrepancies in quality assessment were resolved by consensus after discussion among reviewers.

Results

Study Selection

**Figure 1:**
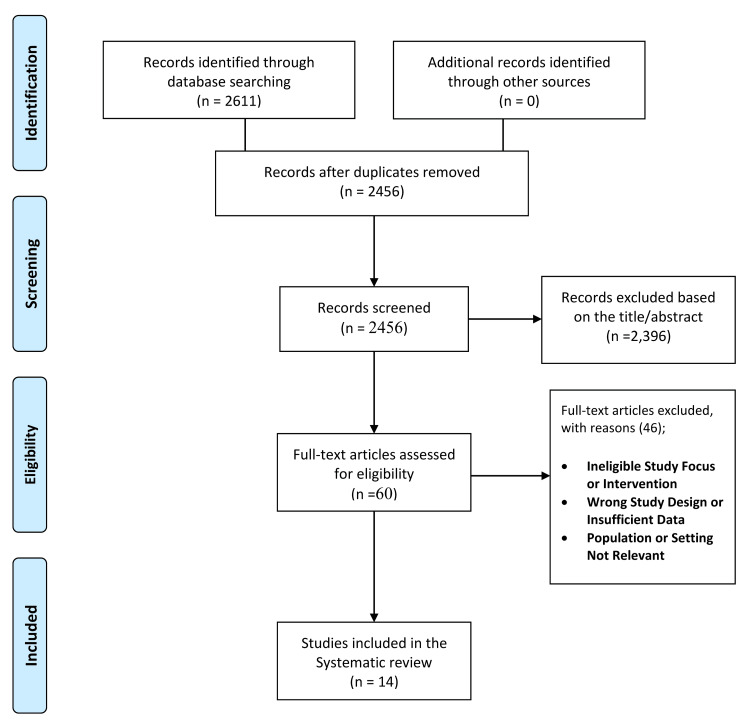
PRISMA flow diagram of study selection process PRISMA: Preferred Reporting Items for Systematic Reviews and Meta-Analyses

A total of 2,611 records were identified through database searching (PubMed: 210; Cochrane: 88; Scopus: 968; WOS: 1,345). No additional studies were retrieved from manual searches of reference lists or grey literature. After removing duplicates, 2,456 unique records remained. Title and abstract screening excluded 2,396 records that did not meet eligibility criteria. Sixty full-text articles were reviewed, of which 46 were excluded for reasons such as ineligible study focus, wrong design, insufficient data, or non-relevant populations/settings. Ultimately, 14 studies met the inclusion criteria. The selection process is illustrated in the PRISMA flow diagram (Figure 1).

Study Characteristics

The 14 included studies were conducted across Belgium, Japan, Thailand, Australia, the USA, Germany, Canada, South Korea, and the UK, representing broad geographical diversity (Table 1). Designs ranged from retrospective comparative analyses to prospective randomized controlled trials, with some employing bilateral within-patient comparisons. Sample sizes ranged from 30 to 200 patients, with mean ages from the mid-60s to mid-70s. Most cohorts had body mass index (BMI) values in the overweight-to-obese range (≈26-36 kg/m²). Gender distribution varied, with some groups predominantly male [6,12] and others female-dominated [13].

**Table 1:** Comparative studies evaluating kinematic, restricted kinematic, functional, and mechanical alignment techniques in total knee arthroplasty aMA: adjusted mechanical alignment; AKSS: American Knee Society Score; BMI: body mass index; cMA/tMA: clinical/tibial mechanical axis; cMPA/mMPTA/mLDFA: coronal mechanical proximal tibial angle/medial mechanical proximal tibial angle/medial lateral distal femoral angle; CPAK: Coronal Plane Alignment of the Knee classification; CR: cruciate-retaining prosthesis; EQ-5D-5L: EuroQol 5-Dimension, 5-Level questionnaire; FJS: Forgotten Joint Score; GRF: ground reaction force; HKA: hip-knee-ankle angle; HSS: Hospital for Special Surgery knee score; ICPD: intercompartmental pressure difference; iKA: inverse kinematic alignment; JLCA/JLO/JLOA: joint line convergence angle/joint line orientation/joint line orientation angle; KJLO: knee joint line orientation; KOOS: Knee Injury and Osteoarthritis Outcome Score; KOOS4: mean of KOOS pain, symptoms, ADL, and QOL subscales; KSS: Knee Society Score; LDFA: lateral distal femoral angle; MCL: medial collateral ligament; MPTA = medial proximal tibial angle; MRI = magnetic resonance imaging; MTPM: maximum total point motion; MUA: manipulation under anesthesia; NS: not significant; OKS: Oxford Knee Score; PASS: patient-acceptable symptom state; PFJ: patellofemoral joint; PROMs: patient-reported outcome measures; PS: posterior-stabilized prosthesis; PSI: patient-specific instrumentation; QOL: quality of life; ROM: range of motion; RSA: radiostereometric analysis; SANE: Single Assessment Numeric Evaluation; SF-36: Short Form 36 Health Survey; TUG: Timed Up and Go test; VAS: visual analogue scale; WOMAC: Western Ontario and McMaster Universities Osteoarthritis Index

Author [Ref]	Country	Design	Patient characteristics (sample size, mean age, BMI, gender)	Prosthesis/technique	Outcome measures used	Results (functional scores, satisfaction, radiographic)	Findings & safety (complications, key findings)
MacDessi et al. [1]	Australia	Randomized controlled trial (superiority, parallel-group)	125 patients (138 knees: KA = 63 pts/70 knees; MA = 62 pts/68 knees); mean age 67.5; BMI ~30; male 59%, female 41%	Posterior-stabilized, fully cemented TKA (Legion, Smith & Nephew); patellar resurfacing; computer-assisted navigation; KA within restrictive safe zone vs MA	Primary: Intraoperative intercompartmental pressure difference (ICPD) via VERASENSE at 10°; secondary: ICPD at 45° and 90°, balanced knees rate, need for balancing procedures, tibiofemoral lift-off; Radiographic alignment; PROMs: KOOS4, FJS-12, EQ-5D-5L	ICPD lower in KA vs MA at 10° (11.7 vs 32.0 psi, p < 0.001), 45° (14.8 vs 25.2, p = 0.004), 90° (11.7 vs 19.1, p = 0.002); KA more often balanced (80% vs 35%, p < 0.001); MA needed more bone recuts (49% vs 9%, p < 0.001); PROMs improved in both groups at 1 yr, no difference	KA improved intraoperative balance and reduced balancing procedures; No increase in malalignment outliers; short-term PROMs similar
Ettinger et al. [2]	Germany	Prospective double-blind randomized controlled trial	98 patients (rKA = 47, MA = 51); mean age 68.8 vs 63.1 (p = 0.018); BMI 28.1 vs 29.0; female ~39%	Medial pivot TKA (GMK Sphere®, Medacta); PSI-guided; rKA restricted within ±3° HKA, ±5° component deviation	KSS (objective, function, satisfaction, expectation), OKS, WOMAC, FJS, radiographic (HKA, mPTA, LDFA, JLO), CPAK classification	At 1 yr: FJS was higher in rKA (62.2 vs 52.4, p = 0.044); KSS satisfaction and expectation were higher in rKA. At 2 yrs: KSS function and satisfaction were higher in rKA; Subgroup: rKA was superior in varus CPAK, no difference in neutral CPAK	One revision in rKA due to tibial malalignment/instability; otherwise, no major complications. rKAis superior for satisfaction, awareness, and function in varus knees
McEwen et al. [8]	Australia	Prospective randomized controlled trial (bilateral, within-patient, computer-assisted)	41 patients (82 knees; KA = 41, MA = 41); Mean age 65 (51–78); BMI 31	CR TKA (Triathlon, Stryker); hybrid fixation; selective patella resurfacing; computer-assisted navigation	PROMs: KOOS, KOOS JR, OKS, FJS; ROM; patient preference; Radiographic (HKA, JLOA, JLCA, PTA); intraop gap balance, releases	At 2 yrs: no difference in KOOS, KOOS JR, OKS, FJS; ROM equal. 51% no preference; among those with preference, more favored KA (14 vs 7, p = 0.03). MA required more releases (p = 0.018). Radiographs: KA more functional JLOA (p = 0.023)	PROMs are equal, but KA is more often preferred and requires fewer releases. Patients are insensitive to mild HKA asymmetry (<7°). Complications: 1 infection revision (KA), 1 arthroscopic release (KA), 2 MUA (bilateral), 1 patellar fracture (MA)
Laende et al. [5]	Canada	Prospective randomized controlled trial	47 patients (KA+PSI = 24, MA+CAS = 23); mean age 64 vs 63; BMI 36 vs 34; female: 16 vs 17	Cemented CR TKA (Triathlon, Stryker) with patellar resurfacing; KA using MRI-based PSI; MA with CAS	Primary: tibial component migration via RSA; secondary: Inducible displacement, OKS, VAS, UCLA, radiographic alignment	Tibial migration was similar at 2 yrs (MTPM 0.40 vs 0.37 mm, p = 0.82). OKS, VAS, UCLA, satisfaction all NS. Radiographs: KA more varus tibial (−3.3° vs −0.8°, p < 0.001)	KA and MA both showed excellent fixation, stability, PROMs, and satisfaction. KA yielded greater tibial varus but no adverse migration. 1 poly exchange in KA; 1 unrelated death (MA)
Dossett et al. [6]	USA	Prospective randomized controlled trial (2-year follow-up)	88 patients (KA = 44, MA = 44); mean age 66; BMI 29 vs 32; predominantly male (95% KA, 88% MA)	Cemented CR TKA with patellar resurfacing (Vanguard, Biomet); KA using MRI-based PSI vs MA with conventional instruments	OKS, WOMAC, KSS, combined KSS, ROM, Radiographic alignment, perioperative outcomes, reoperation	At 2 yrs: OKS 40 vs 33 (p = 0.005); WOMAC 15 vs 26 (p = 0.005); combined KSS 160 vs 137 (p = 0.005); flexion 121° vs 113° (p = 0.002). Radiographs: KA femoral 2.2° valgus, tibial 2.1° varus	KA superior in pain, function, and ROM. Complications: KA-2 MUA, 1 patellar excision; MA-1 skin slough, 1 revision. Similar reoperation rates
Winnock de Grave et al. [7]	Belgium	Comparative retrospective study (Level III)	80 patients (iKA = 40, aMA = 40); mean age: 69.9 vs 67.4; BMI: 29.2 vs 30.0; female: 60% vs 58%	Robotic-assisted TKA (Stryker Triathlon® CR); restricted inverse kinematic alignment (iKA) vs adjusted mechanical alignment (aMA)	Oxford Knee Score (OKS), VAS satisfaction, PASS thresholds, radiographic HKA/MPTA/mLDFA	OKS: iKA 44.6 vs aMA 42.2 (NS); VAS satisfaction was higher with iKA (9.2 vs 8.5, p = 0.012); PASS was achieved: OKS 98% vs 85%, Satisfaction 80% vs 48%; radiographs: iKA yielded slightly more varus alignment and less femoral external rotation	No intraoperative complications; 1 MUA in the aMA group; no revisions. iKA yielded significantly higher satisfaction and better outcomes in varus knees
Dossett et al. [12]	USA	Double-blind randomized controlled trial	82 patients (KA = 41, MA = 41); mean age 65-66; BMI 29 (KA) vs 33 (MA); predominantly male (95% KA, 85% MA)	Cruciate-retaining TKA with patellar resurfacing (Vanguard, Biomet); Cemented; KA with MRI-based PSI vs MA with conventional instruments	Radiographic (HKA, component angles, joint line); WOMAC, OKS, KSS (function + objective), ROM; Perioperative outcomes	At 6 mo: WOMAC 12 (KA) vs 28 (MA, p < 0.001); Oxford 8 vs 15 (p = 0.001); KSS 90 vs 79 (p = 0.001); Function 84 vs 70 (p = 0.004); Flexion 120° vs 115° (p = 0.043). Radiographs: KA femoral 2.4° valgus, tibial 2.3° varus vs MA. Shorter operative time (−21 min, p < 0.001)	KA improved functional scores and ROM at 6 mo without compromising alignment. Complications: KA-1 hematoma, 2 MUA, 1 patellar subluxation; MA-1 hematoma/skin slough, 1 hematoma evacuation, 1 patella fracture. Early failure risk is similar
Matsumoto et al. [13]	Japan	Prospective randomized controlled trial	60 patients (KA = 30, MA = 30); mean age 75.3 vs 76.1; BMI 26.9 vs 25.5; female 85%	Cruciate-retaining TKA (e-motion, B. Braun Aesculap or Persona, Zimmer); Navigation-assisted	2011 Knee Society Score (objective, satisfaction, expectations, functional activities), ROM (flexion/extension), radiographic (HKA, joint line orientation, cMA, tMA)	Flexion significantly better in KA (122.3° vs 116.8°, p = 0.0496); functional KSS higher in KA (68.3 vs 64.0, p = 0.03); objective KSS similar (92.9 vs 92.5); Satisfaction/expectation not significantly different; Radiographs: KA knees more parallel to the floor joint line, more central weight-bearing in tMA	No PFJ complications (no clunk, fracture, subluxation). KA provided superior flexion and functional outcomes, with more physiologic joint line orientation
Yeo et al. [14]	South Korea	Prospective randomized controlled trial	60 patients (KA = 30, MA = 30); mean age 72–74; BMI 26.1 vs 26.9; male/female: 5/25 vs 3/27	Robotic-assisted TKA (ROBODOC, Zimmer NexGen CR); Randomized to KA (2° varus tibial, 2° valgus femoral) vs MA (perpendicular cuts)	HSS, WOMAC, KSS pain + function, ROM; radiographic alignment; varus/valgus laxity; Gait analysis	At ~8 yrs: no difference in HSS, WOMAC, KSS, ROM. Radiographs: KA more natural joint line. Gait: KA more natural varus (−2.6° vs −5.0°, p = 0.03), lower mediolateral GRF (0.01 vs 0.03, p = 0.01)	KA and MA both have excellent long-term outcomes. KA reproduced a more natural gait. No significant complications
Calliess et al. [15]	Germany	Prospective randomized controlled trial	200 patients (KA = 100, MA = 100); KA: 61F/39M, mean age 67 ± 8, BMI 30 ± 4; MA: 57F/43M, mean age 70 ± 8, BMI 30 ± 5	CR TKA (Triathlon, Stryker); KA using PSI (MRI-based); MA using conventional	WOMAC, KSS, radiographic (mMPTA, mLDFA, tibial slope, limb alignment, femoral flexion)	At 12 mo: KSS higher in KA (190 vs 178, p = 0.02); WOMAC lower in KA (13 vs 26, p = 0.001). Radiographs: KA 1° valgus vs MA 1° varus; More outliers in KA with deviation from plan	Complications: KA-2 revisions for instability; MA-1 revision. KA is superior on average but has broader variability. PSI limitations noted
Dossett et al. [16]	USA	Prospective randomized controlled trial (13-year follow-up of original RCT)	88 TKAs (KA = 44, MA = 44); At 13 yrs: 62 patients available; mean age ~66 at baseline; predominantly male VA population	Cemented CR TKA (single manufacturer, patella resurfaced); KA with MRI-based PSI; MA with conventional	PROMs: WOMAC, OKS, KOOS Jr, FJS, Modified-SANE, satisfaction; Radiographic: CT-based angles, HKA, JLO	At 13 yrs: no difference in reoperations/revisions (5 total: 2 KA, 3 MA). PROMs similar, satisfaction higher trend in KA (96% vs 82%, p = 0.16). Radiographs: KA more valgus femur, varus tibia, oblique JLO, HKA similar	Both KA and MA had excellent 13-year survivorship; satisfaction was high, trend was higher in KA. Patellar complications are slightly higher in KA (11% vs 7%)
Chompoosang et al. [17]	Thailand	Prospective randomized controlled trial (bilateral, within-patient)	30 patients (60 knees; FA = 30, MA = 30); mean age 67.9 ± 6.5; BMI 27.7 ± 5.0; female 86.7%	Robotic-assisted bilateral TKA (Mako system, Stryker Triathlon CR, cemented, no patellar resurfacing); One knee randomized to FA, contralateral to MA	Forgotten Joint Score (FJS), KOOS, ROM (flexion), patient satisfaction (VAS), VAS pain (early postop), radiographic (HKA, KJLO, LDFA, MPTA, ankle parameters), soft tissue release	FJS higher in FA at 3 mo (53.3 vs 46.0, p = 0.015) and 6 mo (67.8 vs 57.8, p < 0.001); KOOS slightly higher in FA (NS); ROM better at 1 mo (110.1 vs 104.5, p = 0.042); Satisfaction higher in FA (84.3 vs 79.2, p = 0.001); radiographs: similar HKA; FA knees had joint line more parallel to floor (3.0° vs 4.7°, p < 0.001); In CPAK type I knees, FA superior in knee/ankle alignment and outcomes	FA required less soft tissue release (23.3% vs 76.7%, p < 0.001); no MCL release in FA vs 6.7% in MA; No revisions or major complications
Waterson et al. [18]	UK	Prospective blinded randomized controlled trial	71 patients randomized (KA = 36, MA = 35); mean age ~69; OA; exclusions: >10° deformity, >20° contracture, inflammatory arthritis	Cemented CR TKA (Stryker Triathlon); KA with MRI-based PSI vs MA conventional	KOOS, AKSS, ROM, Functional tests (walk, TUG, stairs, torque), EQ-5D, SF-36, radiographic HKA	At 1 yr: No significant difference in KOOS, EQ-5D, ROM, function. AKSS favored KA at 6 weeks but equalized by 1 year. Quad torque better in KA early but equalized	KA had similar functional outcomes to MA at 1 year; earlier quad recovery trend in KA. No major complications; 1 exclusion (extensor rupture)
Matsumoto et al. [19]	Japan	Prospective quasi-randomized controlled trial	60 patients (KA = 30, MA = 30); mean age 74 vs 76; BMI 26.4 vs 26.2; female ~83%	CR TKA (e-motion, B. Braun Aesculap); Modified KA with restricted tibial cut (3° varus, 7° slope); navigation-assisted	Intraop kinematics (rotation, translation); Soft tissue balance (laxity, gap); ROM; KSS (objective, satisfaction, function); radiographic alignment	KA preserved greater tibial internal rotation (21.1° vs 15.8°, p = 0.03), higher flexion gain (+8.8° vs +3.3°, p = 0.037); KSS improvement greater in KA. Radiographs: KA knees more varus	Modified KA improved kinematics, satisfaction, flexion, KSS. No major complications

Surgical approaches and implant choices differed across trials. Several studies used cruciate-retaining designs [8,12,13], while others employed posterior-stabilized prostheses [1]. Fixation techniques included cemented, hybrid, or fully cemented methods. Patellar resurfacing was either selective or routine. Advanced technologies were increasingly used: robotic-assisted TKA [1,14], computer-assisted navigation [1,5], and MRI-based patient-specific instrumentation [6,12,15]. These reflect the evolving integration of robotics and navigation in optimizing KA versus MA.

Outcome measures were heterogeneous. Common patient-reported measures included the OKS, WOMAC, KSS, KOOS, FJS, and EuroQol 5-Dimension (EQ-5D), with some studies reporting satisfaction and expectation scales. Functional assessments included range of motion, gait analysis, and performance-based tests such as timed up-and-go and stair climbing. Radiographic parameters included hip-knee-ankle angle, MA and KA variables, and joint line orientation. Some studies additionally assessed intraoperative balancing (e.g., intercompartmental pressure differences [1]) or tibial component migration by radiostereometric analysis [5]. Long-term outcomes such as implant survivorship and reoperation rates were evaluated in studies, including Dossett et al. [16].

Quality Assessment

Methodological quality, assessed with the Downs and Black checklist, ranged from 22 to 25 out of 28, indicating moderate-to-high-quality overall. Reporting was consistently strong (9-10/10), demonstrating clear objectives, outcomes, interventions, and findings. External validity varied slightly, with most trials scoring 2/3, while Winnock de Grave et al. [7], Chompoosang et al. [17], and Ettinger et al. [2] achieved 3/3, reflecting better generalizability (Table 2).

**Table 2:** Methodological quality assessment of included studies using the Downs and Black checklist

Study ID	Reporting (0-10)	External validity (0-3)	Internal validity-bias (0-7)	Internal validity-confounding (0-6)	Power (0-1)	Total (0-28)
MacDessi et al. [1]	10	2	6	5	1	24
Ettinger et al. [2]	10	3	6	5	1	25
Laende et al. [5]	10	2	6	5	1	24
Dossett et al. [6]	10	2	6	5	1	24
Winnock de Grave et al. [7]	9	3	5	4	1	22
McEwen et al. [8]	10	2	6	5	1	24
Dossett et al. [12]	9	2	6	5	1	23
Matsumoto et al. [13]	9	2	6	5	1	23
Yeo et al. [14]	10	2	6	5	1	24
Calliess et al. [15]	9	2	6	5	1	23
Dossett et al. [16]	9	2	6	5	1	23
Chompoosang et al. [17]	10	3	6	5	1	25
Waterson et al. [18]	10	2	6	5	1	24
Matsumoto et al. [19]	9	2	6	5	1	23

Internal validity regarding bias was consistently high (5-6/7), and confounding was appropriately addressed (≈5/6). Power was uniformly scored as 1, suggesting sample sizes were generally adequate for detecting clinically meaningful differences. The highest overall quality (25/28) was reported in Chompoosang et al. [17] and Ettinger et al. [2], followed by several studies scoring 24/28 [1,5,8,14,18]. The lowest score, 22/28, was observed in Winnock de Grave et al. [7], though still within the moderate-to-high range. Collectively, these findings confirm that most included studies were well designed, adequately reported, and appropriately powered.

Effect of Intervention on OKS

KA generally demonstrated early-to-midterm advantages. Dossett et al. [6,12] reported ≈7-point gains favoring KA, while Winnock de Grave et al. [7] showed higher rates of Patient Acceptable Symptom State achievement with restricted inverse kinematic alignment (iKA), especially in varus knees. Conversely, Laende et al. [5] and McEwen et al. [8] reported no group differences at two years, likely due to ceiling effects. Ettinger et al. [2] found that benefits were strongest in varus phenotypes. At 13 years, Dossett et al. [6] found no differences, suggesting convergence over time.

Effect of Intervention on WOMAC

KA demonstrated clear short-term benefits in pain and function domains. Dossett et al. [6,12] and Calliess et al. [15] reported large differences within 24 months, while Ettinger et al. [2] confirmed restricted KA advantages in varus phenotypes. Yeo et al. [14] and Laende et al. [5] reported equivalence, and Dossett et al. [16] found no long-term difference. Benefits appear most evident early, diminishing as both groups plateau at high function.

Effect of Intervention on KSS

KA was frequently associated with higher functional and satisfaction scores. Dossett et al. [12,16] and Calliess et al. [15] demonstrated superiority at 12 months, while Matsumoto et al. [13,19] reported better flexion and satisfaction under KA. Ettinger et al. [2] confirmed restricted KA advantages, strongest in varus phenotypes. Waterson et al. [18] noted only transient early benefits.

Effect of Intervention on FJS

KA often produced higher values, reflecting greater “joint forgettability.” Chompoosang et al. [17] and Ettinger et al. [2] reported significantly higher scores at 3-12 months, especially in varus subgroups. McEwen et al. [8] found no mean differences but noted that more patients preferred their KA knee.

Effect of Intervention on KOOS

Findings were mixed. McEwen et al. [8] found equivalence, while Chompoosang et al. [17] and Ettinger et al. [2] observed directional advantages for KA, most notable in varus phenotypes.

Effect of Intervention on Range Of Motion

KA was consistently associated with improved flexion in the short term. Dossett et al. [6,12] reported +5° to +8.5° gains, and Matsumoto et al. [13,19] showed superior flexion recovery. Chompoosang et al. [17] observed earlier flexion gains, while Yeo et al. [14] and Waterson et al. [18] found no long-term differences.

Effect of Intervention on Patient Satisfaction

Satisfaction generally favored KA, particularly in varus phenotypes. Winnock de Grave et al. [7], Chompoosang et al. [17], and Ettinger et al. [2] reported higher early satisfaction and Patient Acceptable Symptom State achievement, while McEwen et al. [8] noted patient preference for KA of knees. Long-term data from Dossett et al. [16] showed no significant difference.

Effect of Intervention on Radiographic Alignment and Joint Line Orientation

KA consistently restored more valgus femoral and varus tibial positions with joint lines closer to native orientation. Dossett et al. [6,12,16] quantified these changes. Laende et al. [5] and Yeo et al. [14] confirmed more anatomic coronal angles and more physiologic gait patterns under KA.

Effect of Intervention on Soft-Tissue Balance

KA was associated with fewer releases and improved intraoperative balance. MacDessi et al. [1], Chompoosang et al. [17], and McEwen et al. [8] all reported fewer interventions required to achieve balance, reflecting closer reproduction of native ligament tension.

Effect of Intervention on Complications and Revisions

Complication and revision rates were low and comparable between groups. Calliess et al. [15] noted slightly higher early instability revisions with KA, linked to technical outliers. Long-term survivorship was equivalent in Dossett et al. [16]. Other studies [5,8,14] also found no significant differences, confirming that KA remains safe within restricted boundaries.

## Conclusions

KA in TKA appears to provide meaningful short- to mid-term advantages over MA, particularly in terms of functional outcomes, joint awareness, patient satisfaction, and early range of motion while maintaining comparable long-term survivorship and complication rates. These benefits are most consistently observed in varus phenotypes and when alignment is performed within restricted, phenotype-aware boundaries that respect native joint line orientation. Although methodological heterogeneity limits universal conclusions, the accumulated evidence supports KA as a safe and effective alternative to MA, particularly for patients seeking faster recovery and a more natural-feeling joint, while underscoring the need for high-quality, long-term, phenotype-stratified randomized trials.

## References

[1] MacDessi SJ, Griffiths-Jones W, Chen DB, Griffiths-Jones S, Wood JA, Diwan AD, Harris IA (2020). Restoring the constitutional alignment with a restrictive kinematic protocol improves quantitative soft-tissue balance in total knee arthroplasty: a randomized controlled trial. Bone Joint J.

[2] Ettinger M, Tuecking LR, Savov P, Windhagen H (2024). Higher satisfaction and function scores in restricted kinematic alignment versus mechanical alignment with medial pivot design total knee arthroplasty: a prospective randomised controlled trial. Knee Surg Sports Traumatol Arthrosc.

[3] Matassi F, Pettinari F, Frasconà F, Innocenti M, Civinini R (2023). Coronal alignment in total knee arthroplasty: a review. J Orthop Traumatol.

[4] Bellemans J, Colyn W, Vandenneucker H, Victor J (2012). The Chitranjan Ranawat award: is neutral mechanical alignment normal for all patients? The concept of constitutional varus. Clin Orthop Relat Res.

[5] Laende EK, Richardson CG, Dunbar MJ (2019). A randomized controlled trial of tibial component migration with kinematic alignment using patient-specific instrumentation versus mechanical alignment using computer-assisted surgery in total knee arthroplasty. Bone Joint J.

[6] Dossett HG, Estrada NA, Swartz GJ, LeFevre GW, Kwasman BG (2014). A randomised controlled trial of kinematically and mechanically aligned total knee replacements: two-year clinical results. Bone Joint J.

[7] Winnock de Grave P, Luyckx T, Claeys K, Tampere T, Kellens J, Müller J, Gunst P (2022). Higher satisfaction after total knee arthroplasty using restricted inverse kinematic alignment compared to adjusted mechanical alignment. Knee Surg Sports Traumatol Arthrosc.

[8] McEwen PJ, Dlaska CE, Jovanovic IA, Doma K, Brandon BJ (2020). Computer-assisted kinematic and mechanical axis total knee arthroplasty: a prospective randomized controlled trial of bilateral simultaneous surgery. J Arthroplasty.

[9] Page MJ, McKenzie JE, Bossuyt PM (2021). The PRISMA 2020 statement: an updated guideline for reporting systematic reviews. BMJ.

[10] Schardt C, Adams MB, Owens T, Keitz S, Fontelo P (2007). Utilization of the PICO framework to improve searching PubMed for clinical questions. BMC Med Inform Decis Mak.

[11] Downs SH, Black N (1998). The feasibility of creating a checklist for the assessment of the methodological quality both of randomised and non-randomised studies of health care interventions. J Epidemiol Community Health.

[12] Dossett HG, Swartz GJ, Estrada NA, LeFevre GW, Kwasman BG (2012). Kinematically versus mechanically aligned total knee arthroplasty. Orthopedics.

[13] Matsumoto T, Takayama K, Ishida K, Hayashi S, Hashimoto S, Kuroda R (2017). Radiological and clinical comparison of kinematically versus mechanically aligned total knee arthroplasty. Bone Joint J.

[14] Yeo JH, Seon JK, Lee DH, Song EK (2019). No difference in outcomes and gait analysis between mechanical and kinematic knee alignment methods using robotic total knee arthroplasty. Knee Surg Sports Traumatol Arthrosc.

[15] Calliess T, Bauer K, Stukenborg-Colsman C, Windhagen H, Budde S, Ettinger M (2017). PSI kinematic versus non-PSI mechanical alignment in total knee arthroplasty: a prospective, randomized study. Knee Surg Sports Traumatol Arthrosc.

[16] Dossett HG, Arthur JR, Makovicka JL, Mara KC, Bingham JS, Clarke HD, Spangehl MJ (2023). A randomized controlled trial of kinematically and mechanically aligned total knee arthroplasties: long-term follow-up. J Arthroplasty.

[17] Chompoosang T, Ketkaewsuwan U, Ploynumpon P (2025). Comparative effects of mechanical and functional alignment in bilateral robotic total knee arthroplasty: a randomized controlled trial. Arthroplasty.

[18] Waterson HB, Clement ND, Eyres KS, Mandalia VI, Toms AD (2016). The early outcome of kinematic versus mechanical alignment in total knee arthroplasty: a prospective randomised control trial. Bone Joint J.

[19] Matsumoto T, Takayama K, Ishida K (2020). Intraoperative soft tissue balance/kinematics and clinical evaluation of modified kinematically versus mechanically aligned total knee arthroplasty. J Knee Surg.

